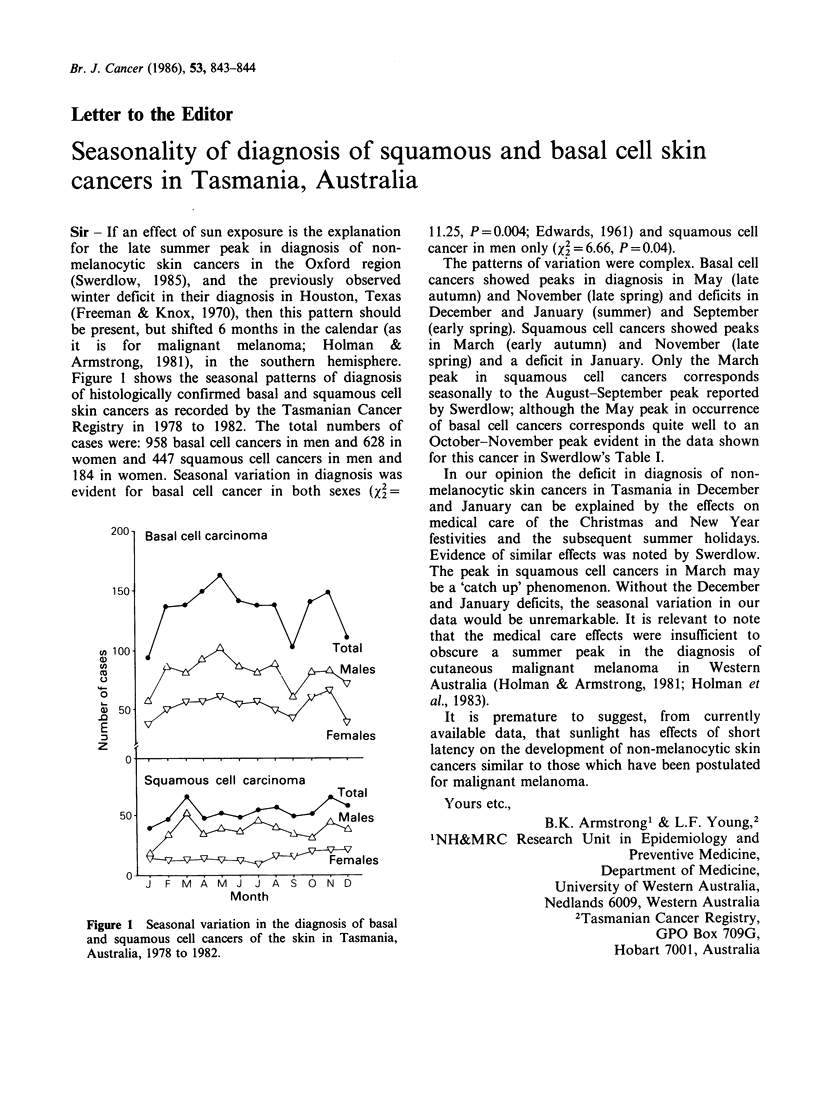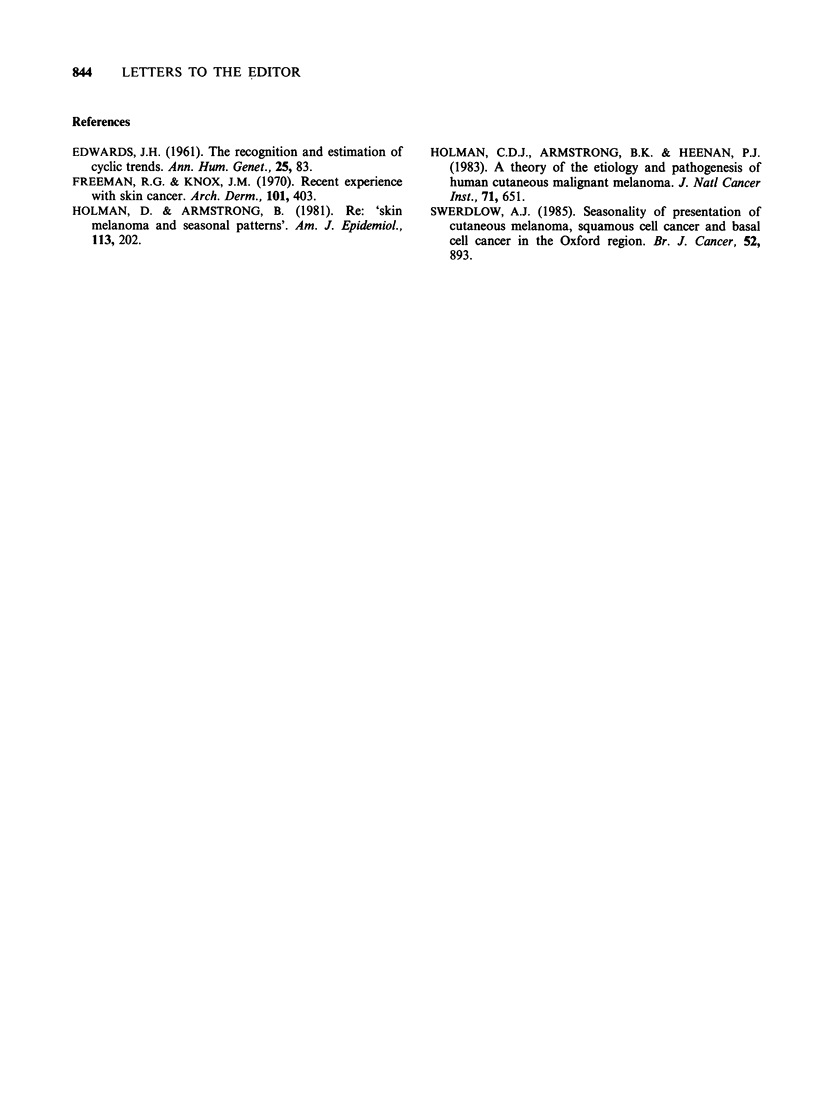# Seasonality of diagnosis of squamous and basal cell skin cancers in Tasmania, Australia.

**DOI:** 10.1038/bjc.1986.143

**Published:** 1986-06

**Authors:** B. K. Armstrong, L. F. Young


*Br. J. Cancer* (1986), **53**, 843–844

**Letter to the Editor**

### Seasonality of diagnosis of squamous and basal cell skin cancers in Tasmania, Australia

**Sir** – If an effect of sun exposure is the explanation for the late summer peak in diagnosis of non-melanocytic skin cancers in the Oxford region (Swerdlow, 1985), and the previously observed winter deficit in their diagnosis in Houston, Texas (Freeman & Knox, 1970), then this pattern should be present, but shifted 6 months in the calendar (as it is for malignant melanoma; Holman & Armstrong, 1981), in the southern hemisphere. Figure 1 shows the seasonal patterns of diagnosis of histologically confirmed basal and squamous cell skin cancers as recorded by the Tasmanian Cancer Registry in 1978 to 1982. The total numbers of cases were: 958 basal cell cancers in men and 628 in women and 447 squamous cell cancers in men and 184 in women. Seasonal variation in diagnosis was evident for basal cell cancer in both sexes ($\chi_2^2 = 11.25$, $P = 0.004$; Edwards, 1961) and squamous cell cancer in men only ($\chi_2^2 = 6.66$, $P = 0.04$).

**Figure 1** Seasonal variation in the diagnosis of basal and squamous cell cancers of the skin in Tasmania, Australia, 1978 to 1982.

The patterns of variation were complex. Basal cell cancers showed peaks in diagnosis in May (late autumn) and November (late spring) and deficits in December and January (summer) and September (early spring). Squamous cell cancers showed peaks in March (early autumn) and November (late spring) and a deficit in January. Only the March peak in squamous cell cancers corresponds seasonally to the August–September peak reported by Swerdlow; although the May peak in occurrence of basal cell cancers corresponds quite well to an October–November peak evident in the data shown for this cancer in Swerdlow's Table I.

In our opinion the deficit in diagnosis of non-melanocytic skin cancers in Tasmania in December and January can be explained by the effects on medical care of the Christmas and New Year festivities and the subsequent summer holidays. Evidence of similar effects was noted by Swerdlow. The peak in squamous cell cancers in March may be a 'catch up' phenomenon. Without the December and January deficits, the seasonal variation in our data would be unremarkable. It is relevant to note that the medical care effects were insufficient to obscure a summer peak in the diagnosis of cutaneous malignant melanoma in Western Australia (Holman & Armstrong, 1981; Holman *et al.*, 1983).

It is premature to suggest, from currently available data, that sunlight has effects of short latency on the development of non-melanocytic skin cancers similar to those which have been postulated for malignant melanoma.

Yours etc.,

B.K. Armstrong[1] & L.F. Young,[2]
[1]NH&MRC Research Unit in Epidemiology and Preventive Medicine,
Department of Medicine,
University of Western Australia,
Nedlands 6009, Western Australia
[2]Tasmanian Cancer Registry,
GPO Box 709G,
Hobart 7001, Australia

**References**

EDWARDS, J.H. (1961). The recognition and estimation of cyclic trends. *Ann. Hum. Genet.*, **25**, 83.

FREEMAN, R.G. & KNOX, J.M. (1970). Recent experience with skin cancer. *Arch. Derm.*, **101**, 403.

HOLMAN, D. & ARMSTRONG, B. (1981). Re: 'skin melanoma and seasonal patterns'. *Am. J. Epidemiol.*, **113**, 202.

HOLMAN, C.D.J., ARMSTRONG, B.K. & HEENAN, P.J. (1983). A theory of the etiology and pathogenesis of human cutaneous malignant melanoma. *J. Natl Cancer Inst.*, **71**, 651.

SWERDLOW, A.J. (1985). Seasonality of presentation of cutaneous melanoma, squamous cell cancer and basal cell cancer in the Oxford region. *Br. J. Cancer*, **52**, 893.